# In silico study of a bilayer titanium dental implant with a porous titanium and hydroxyapatite composite outer layer for enhanced osseointegration

**DOI:** 10.1038/s41598-025-31030-0

**Published:** 2026-01-12

**Authors:** Vamsi Krishna Dommeti, Francesco Valente, Cristina Falcinelli, Tonino Traini, Goldina Ghosh, Sandipan Roy

**Affiliations:** 1 Department of Mechanical Engineering, College of Engineering and Technology, Faculty of Engineering and Technology, SRM Institute of Science and Technology, Kattankulathur, Kanchipuram, Chennai, Tamil Nadu India; 2 Department of Innovative Technologies in Medicine & Dentistry, University “G. d’Annunzio” of Chieti-Pescara, Via Dei Vestini 31, 66100 Chieti, Italy; 3 Electron Microscopy Laboratory, University “G. d’Annunzio” of Chieti-Pescara, Via Dei Vestini 31, 66100 Chieti, Italy; 4 Department of Engineering and Geology, University “G. d’Annunzio” of Chieti-Pescara, Viale Pindaro 42, 65127 Pescara, Italy; 5 Computer Applications & Science, Institute of Engineering and Management, Kolkata, 700091 India

**Keywords:** Bilayered dental implant, Porous titanium implants, Hydroxyapatite dental implants, Finite element analysis, HA composite, Bone-implant interface, Stress analysis, Biomedical engineering, Dental implants

## Abstract

This study aims to develop an innovative bilayered dental implant design featuring a titanium alloy core with a porous composite titanium (Ti) and hydroxyapatite (HA) outer layer to enhance implant stability and patient outcomes. Using SolidWorks 2017, 3D models of the implants and a mandibular bone segment were created. A Finite Element (FE) analysis was then conducted with ANSYS Workbench to assess the mechanical behavior under a 250 N axial compressive load, comparing the bilayered implant to a conventional titanium implant. Variables like porosity (ranging from 10 to 90% in 10% increments), HA content (ranging from 10 to 50% in 5% intervals), and outer layer thickness (2 mm, 1.5 mm and 1 mm) were systematically analyzed. Each configuration was evaluated based on von Mises stress distribution and interfacial strain in peri-implant bone. Results indicated that all porous designs of bilayered implants had significantly lower von Mises stress than traditional implant, with reductions ranging from approximately 69 to 94%, depending on HA/Ti composition and shell thickness. The non-porous bilayer configurations also showed clear stress reductions, with decreases from approximately 72 to 90%, depending on the HA/Ti composition and shell thickness. However, these reductions were slightly lower than those observed in porous designs, with maximal reductions occurring in the porous core of some 2 mm bilayered implant configurations. The combined evaluation of strain and von Mises stress analyses identified the 2 mm core diameter with a 2 mm porous shell as the optimal design, providing favorable microstrain, improved load transfer, and reduced stress concentrations. This modification promotes a more favorable mechanical interaction between the implant and surrounding bone. These findings underscore the potential of bilayered porous implants to improve stability and bone integration, marking a significant step forward in dental implant technology. Further research, including experimental validation, is encouraged to verify these results and investigate other loading conditions, promoting the development of more effective and sustainable dental implant solutions.

## Introduction

Dental implants are commonly used to support and/or retain prostheses for restoring completely or partially edentulous patients. The long-term success of dental implants depends on both osseointegration and interfacial load transfer between implants and surrounding bone tissue. Osseointegration is a complex process involving cellular and molecular interactions for bone remodeling at the interface, influenced by factors such as primary implant stability, surrounding bone quality and quantity, implant geometry and surface topography, surgical practices, potential infection, and patient’s overall and local health conditions^1–4^. In the field of dental implantology the achieving of a complete osseointegration is still highly investigated by both dentists and biomedical engineers through the development of implant solutions and strategies that may enhance the osseointegration avoiding possible implant failure. Occlusal overload is one of the most important factors associated with late failure^4^, highlighting the critical role of stress transfer from the implant to the surrounding bone. To identify potential risks and improve clinical outcomes, it is essential to use numerical methods, such as finite element modelling and analysis, which is widely utilized in dentistry^5^. The stress transfer essentially depends on material properties, implant geometry, prosthesis type, and loading conditions^6^. While the loading conditions are unmodifiable, the optimization of the implant surface and material properties can facilitate effective load transmission and promote better osseointegration.

In terms of surface properties, implants have been designed with modifications at both the micro-level and macro level^7^. At the micro-level, surface modifications like sandblasting, acid etching, micro-arc oxidation, and hydroxyapatite (HA) coating have been introduced to enhance mechanical interlocking by introducing surface irregularities, potentially increasing bone-implant contact, influencing biomaterial-tissue interactions, and improving osteoinductivity and osseointegration^8^. At the macro-level, modern dental implants often incorporate design elements like grooves, pitches, fins, and pores to augment the contact surface with bone and enhance biological anchorage. The overall geometry of these implants plays a crucial role in their primary stability and the distribution of stress^9^.

In terms of material properties, titanium (Ti) alloys, in particular Ti6Al4V, are the preferred materials in implant fabrication due to the combination of their biocompatibility, corrosion resistance and mechanical properties^10^. However, Ti6Al4V presents some drawbacks. First, its Young’s modulus is around 105–120 GPa, which is higher than the human cortical bone (around 15–20 GPa). This disparity in stiffness can result in the "stress shielding effect," a well-known phenomenon in biomechanics. This occurs due to shear stresses caused by the disparity in elastic modulus between bone and the implant, which may lead to bone resorption, implant loosening, and fracture^11^. Second, Ti6Al4V contains alloying elements like aluminium (Al) and vanadium (V) that may produce toxic effects in body fluids^12^. To address these mechanical incompatibilities, various strategies have been explored, such as using titanium alloys with a lower Young’s modulus, like beta titanium^13^, or the development of porous implants that allow to reduce the Young’s modulus based on the amount and size of the pores and thus provide internal bone growth favouring mechanical imbrication^13^, or varying the density and stiffness with a gradient of porosity perpendicular to the long axis of the implant produced via additive techniques^14^. Improving implant bioactivity to promote better osseointegration and reduce the risk of stress shielding is crucial. Hydroxyapatite, a bioactive material that promotes bone growth and stronger bone-implant bonding, is commonly used for coating implants^15,16^. However, the stability of HA coatings has been observed to be problematic with conventional techniques, such as plasma spraying and perfusion electrodeposition methods, and can only be observed for a short time^17^. These techniques lead to a weak bonding strength between HA coating and metal substrate, resulting in separation^18^. Powder metallurgy is used to create a composite of HA and titanium alloy, addressing these stability issues and retaining the bioactive benefits of HA^19–21^. However, the high elastic modulus of HA particles can affect stress transfer to the bone due to stiffness mismatching^22–24^. To overcome these limitations in the work performed by Raj et al. a novel three-layered implant design has been proposed to address this issue^25^. This design consists of a low modulus, high strength titanium alloy core, an outer layer incorporating HA, and a porous titanium alloy layer between the core and the outer layer demonstrating improved osseointegration and stress distribution. Based on this concept and in contrast to previous strategies based on surface coatings, composite materials, or complex multilayered designs, the present study introduces a bilayered dental implant composed of a Ti alloy core and a porous titanium outer layer incorporating HA. This design aims to combine mechanical strength with bioactivity, enhancing bone-implant integration while reducing stress shielding.

Recent literature supports the rationale for such hybrid designs. Bilayered ceramic prostheses (e.g., zirconia–lithium disilicate) have shown high survival rates and reduced prosthetic complications in posterior restorations, suggesting improved mechanical reliability and stress distribution^26^. Moreover, bilayered scaffolds have demonstrated enhanced bone regeneration and mechanical compatibility^27^. Recent studies on functionally graded dental implants (FGMs) have shown that a gradual transition in material properties from a titanium core to a bioactive outer layer can significantly improve bone remodeling and stress distribution. Jafari et al.^28^ demonstrated through finite element simulations that radial FGM implants produced higher bone density and lower stress concentrations compared to conventional titanium and HA-coated implants. Although FGMs differ from bilayered designs in their continuous gradient, both approaches share the principle of combining mechanical strength with bioactivity to enhance osseointegration.

The implant proposed in the present study consists of a Ti alloy core and an outer porous titanium layer incorporating HA to enhance bone-implant integration. The present work specifically investigates the effect of three key design parameters: (1) the percentage of porosity in the outer layer, (2) the amount of HA content, and (3) the thickness of the porous outer layer. These variables are analyzed through FE analysis under realistic load conditions to assess their impact on the distribution of mechanical loads and the resulting von Mises stress in the surrounding bone tissue, and the results are compared with those obtained for a conventional implant. The novelty of this work lies in the development of an innovative bilayered design and in the systematic investigation of three design parameters through FE analysis to identify their combined effect on load transfer and stress distribution. This provides a parametric framework to optimize biomechanical compatibility, aiming to reduce stress shielding while enhancing osseointegration. Moreover, by directly comparing the bilayered design with a conventional implant, the study demonstrates its potential to achieve improved primary stability and long-term clinical performance with a simpler and more practical configuration than previously proposed multilayered solutions.

## Methods

In the following, the approach used in the present work is described. Since this study does not involve human subjects, animal experiments, or sensitive data, no ethical approval was required.

### CAD modelling

The 3D bone domain and dental implants were created by using CAD software’s SolidWorks 2017 (Dassault Systems, USA). In detail, a 3D CAD model of a mandibular section was created, 24.2 mm in height and 16.3 mm in width, consisting of a cancellous bone core surrounded by a 2-mm cortical bone^29^. Simplified CAD models of monolithic dental implants were designed. For all CAD models the fixture has been characterized by a diameter of 6 mm and a length of 13 mm. The general shape consisted of a cylindrical implant, bottom straight, with a single, continuous triangular thread of 1 mm and a constant thread pitch of 1 mm according to Kong et al.^30^. A cap has been added to the superior part of all CAD models^31^.

**Fig. 1:**
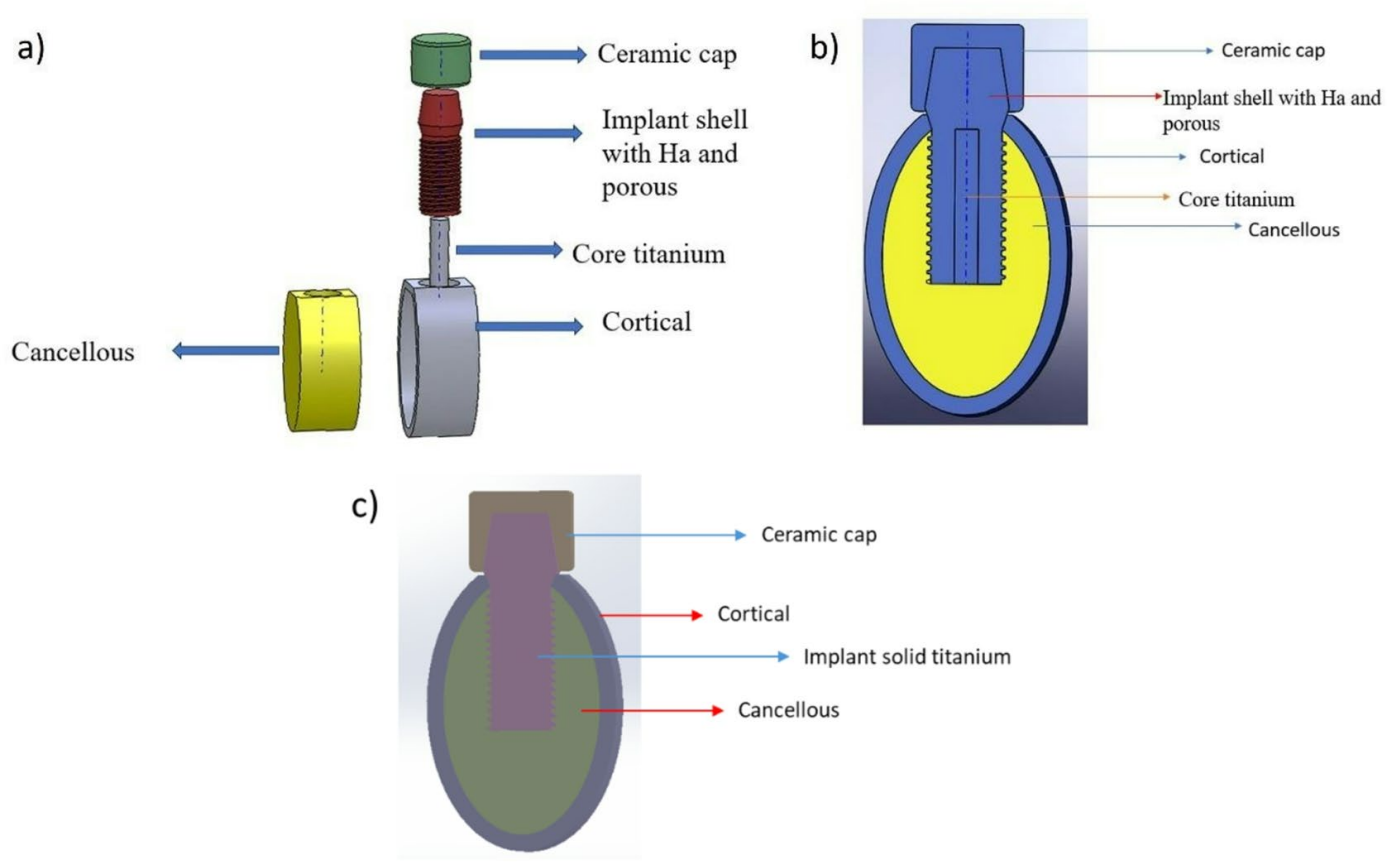
CAD models of the bilayer and conventional implants designed and investigated in the present study. (**a**) Whole model with all parts disassembled (bilayer implant). (**b**) Section parallel to the long axis of the bilayer implant showing all the parts assembled. (**c**) Conventional solid implant.

The CAD model was designed with a diameter of 6 mm and with an external porous shell and an inner core fully solid. The external shell has been characterized by the thickness T_L_ for which three different values have been investigated, i.e. 2 mm, 1.5 mm and 1 mm, leading to realizing three different implant CAD models. Depending on the values of T_L_, the diameter of the inner core (d) assumes the following values: 2 mm (when T_L_ = 2 mm), 3 mm (when T_L_ = 1.5 mm) and 4 mm (when T_L_ = 1 mm). This kind of implant will be identified in the following as bilayer implant. In what follows the “T_L_ value bilayer” notation will be adopted for brevity, so “2 mm bilayer” stands for “a bilayer implant with a thickness of the external layer equal to 2 mm”. Figure 1a and b show the bilayer implant with all its parts and the bone segment. The other type of implant CAD model was designed as a fully solid monolithic implant without porosity (Fig. 1c). This implant will be identified in the following as “conventional”.

### Computational discretization

Once the CAD models have been assembled, they have been imported into the Ansys Workbench FE analysis tool, v. 18.2 (Ansys, Inc., Canonsburg, USA). Ten-node tetrahedral elements with quadratic shape functions and 3 degrees of freedom at each node (SOLID 187 elements) have been employed for both bone and dental implants. To ensure suitable accuracy of the numerical FE solutions, mesh size for the bone-implant models was set up as a result of a convergence analysis. In general, during a convergence analysis, the mesh size is progressively reduced, and the resulting parameters—such as stress and strain—are monitored. The mesh is considered adequate when further refinement results in minimal changes to these parameters, indicating reliable and stable results. In the present work, based on the convergence test, the mean element size was set to approximately 0.2 mm. This mesh size allowed the stress values from the finite element model to converge with an accuracy of 3%, which is below the 5% threshold typically used to define a converged mesh.

Table 1 summarizes the number of elements and nodes for the bone-implant FE models analyzed in the present work.

**Table 1:** Number of elements and nodes used in finite element models.

Type of implant	Number of elements	Number of nodes
1 mm bilayer	3,353,320	4,628,408
1.5 mm bilayer	3,018,155	4,266,527
2 mm bilayer	3,321,299	4,587,341
Conventional	3,364,330	4,639,993

### Constitutive behavior and material parameters

The involved materials for both bone and dental implants have been assumed to have a linearly elastic isotropic behavior, and the homogeneity of all materials has been considered. Bone elastic material properties were set as follows in agreement with^32^:

- Poisson’s ratio ν_bone_ of both cortical and trabecular bone equal to 0.30;
- Young’s modulus of the cortical bone E_cort_ equal to 13 GPa;
- Young’s modulus of the trabecular bone E_trab_ equal to 1.37 GPa.

The cap added to the dental implants has been considered made of ceramic with a Poisson’s ratio ν_cap_ of 0.28 and a Young’s modulus E_cap_ of 68.9 GPa. These values were obtained from the literature. Based on the type of dental implant analyzed (i.e., bilayer or conventional) different material properties have been employed as described in detail in the following.

#### Bilayer dental implant

To establish the material properties of bilayer implant the rule of mixture has been used to compute the maximum (upper bound) and minimum (lower bound) Young’s modulus (${E}_{max}$ and ${E}_{min}$, respectively) for the bilayer implant without and with porosity. This rule is a well-established method to identify the properties of each phase using the corresponding volume fraction of each phase to predict the properties of the given material. In detail, assuming a Young modulus of 120 GPa for Ti (${E}_{Ti}$) and 70 GPa for HA (${E}_{HA}$), which were derived from the literature^33,34^, the ${E}_{max}$ and ${E}_{min}$ values for the bilayer implant without porosity have been calculated using the following relations^35^:

$${E}_{max}={E}_{Ti}\cdot {V}_{Ti}+{E}_{HA}\cdot {V}_{HA}$$

$$\frac{1}{{E}_{min}}=\frac{{V}_{Ti}}{{E}_{Ti}}+\frac{{V}_{HA}}{{E}_{HA}}$$

with ${V}_{Ti}$ and ${V}_{HA}$ the volume fractions of Ti and HA, respectively. For a porous bilayer implant the ${E}_{max}$ and ${E}_{min}$ values have been calculated in the following way^35^:

$${E}_{max}=\left(\frac{\%HA}{100}\right)\times {E}_{HA}+\left(\frac{\%Ti}{100}\right)\times [{E}_{Ti}\left(1-\%p)/100\right)]$$

$${E}_{min}=(\frac{\left(\frac{\%HA}{100}\right)}{{E}_{HA}}+\frac{\left(\frac{\%Ti}{100}\right)}{{E}_{Ti}\times \left(1-\%p)/100\right)}{)}^{-1}$$

with $\%p$ the percentage of porosity. In this study, porosity was modeled using a homogenization approach based on the rule of mixtures. The effective Young’s modulus values (${E}_{max}$ and ${E}_{min}$) for the porous HA/Ti composite have been calculated by adjusting the volume fraction of Ti to account for the percentage of porosity. This method allows for the estimation of the mechanical behavior of the porous structure without explicitly modeling the pore geometry, which would require complex stochastic or geometric representations.

Table 2 shows the percentages of Ti, HA and porosity, the Young’s modulus values for Ti and HA and the maximum (${E}_{max}$) and minimum (${E}_{min}$) Young modulus values computed using the rule of mixture. To evaluate the influence of varying HA content and porosity on the mechanical properties of the bilayered implant, we systematically adjusted the HA/Ti ratios and porosity levels in our models. HA content was incrementally increased from 10 to 50% in 5% intervals, with corresponding decreases in titanium content from 90 to 50%. Porosity was varied from 10 to 90% in 10% increments to investigate its influence on implant stiffness and stress distribution. The porosity was selected based on literature recommendations to promote bone ingrowth. Literature reports various optimal pore size ranges for bone formation, including 200–500 μm^36^, ~ 325 μm^37^, 500–1500 μm^38^, and > 100 μm with 75–85% porosity^39^. Previous studies by one of the authors^23,24^ demonstrated that pore sizes of 595 ± 158 μm and 810 ± 172 μm (10% and 20% porosity, respectively) effectively supported cell growth and remodeling. The porosity range adopted in this study falls within these reported values, ensuring favorable conditions for bone ingrowth while enabling mechanical evaluation of the bilayered dental implant. This approach allowed us to investigate how changes in HA content and porosity affect implant stiffness and stress distribution, with the objective of approximating the mechanical properties of natural bone. By combining different HA/Ti ratios with varying porosity levels, we aimed to identify models that optimize the balance between mechanical strength and biological performance, providing adequate support while promoting bone growth and integration.

**Table 2:** Young modulus values for bilayer implant with and without porous composition.

	$\%p$	${E}_{max}$(with porosity) (MPa)	${E}_{min}$(with porosity) (MPa)	${E}_{max}$(without porosity) (MPa)	${E}_{min}$(without porosity) (MPa)
10HA90Ti	10	104,200	102,430	115,000	112,000
15HA85Ti	20	97,200	90,930	112,500	10,830
20HA80Ti	30	90,800	80,760	110,000	105,000
25HA75Ti	40	85,000	71,480	107,500	101,800
30HA70Ti	50	79,800	62,680	105,000	98,800
35HA65Ti	60	75,200	53,930	102,500	96,000
40HA60Ti	70	71,200	44,680	100,000	93,300
45HA55Ti	80	67,800	34,070	97,500	90,800
50HA50TI	90	65,000	20,480	95,000	88,420

In the first column the notation “%HA%Ti” has been adopted to identify the different analyzed cases. For example, 10HA90Ti stands for an implant characterized by 10% of hydroxyapatite and 90% of titanium.

#### Conventional dental implant

The conventional implant has been assumed to be fully constituted of Ti6Al4V with a Poisson’s ratio ν_IC_ and a Young’s modulus E_IC_ of 0.35 and 120 GPa, respectively^32^.

### Boundary conditions

**Fig. 2:**
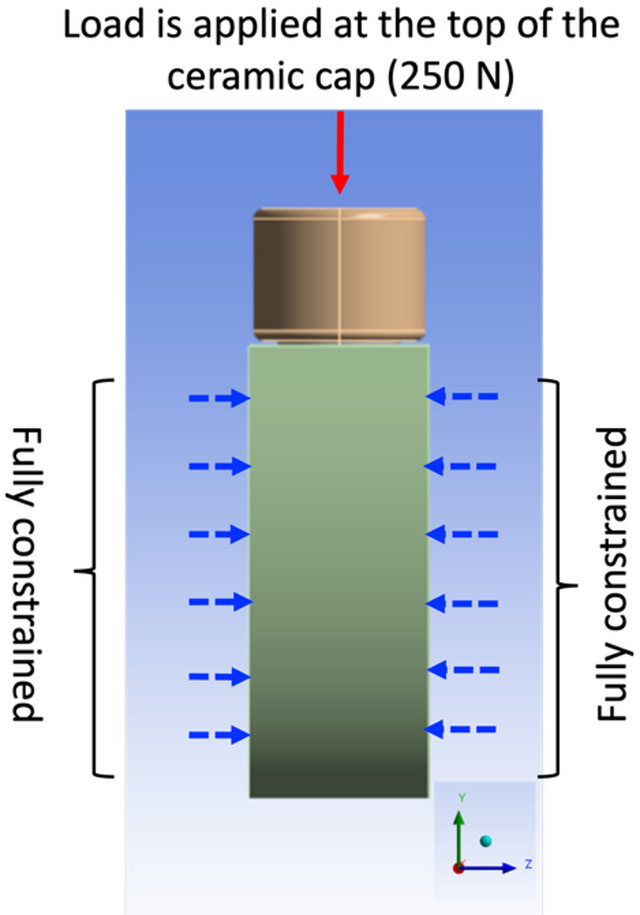
Boundary conditions used in the present work. The red arrow represents a compressive load applied along the y-axis of the reference system over the entire ceramic cap, while the blue arrows denote the bone surfaces that were fully constrained.

Finite element simulations were carried out considering a static axial compressive load of 250 N load applied at the top of the cap to represent the average biting force^5^ (Fig. 2). Moreover, the lateral surfaces of the bone was assumed to be fixed and thus all nodal displacement components were set equal to zero (Fig. 2). A bonded surface-to-surface contact between the implant shell to core titanium bar, and between the implant and the surrounding bone has been considered to simulate a complete osseointegration. In addition, the same type of contact has been used between the cap and dental implant.

### Finite element analysis

The FE analyses have been performed on both conventional and bilayer dental implants. For the bilayer implant three values of shell thickness have been considered (T_L_ = 2, 1.5 and 1 mm). Moreover, for each shell thickness value the ${E}_{max}$ and ${E}_{min}$ values reported in Table 2 have been considered resulting in 54 FE simulations for the case without porosity and 54 FE simulations considering the presence of porosity. This resulted in a total of 109 FE analyses (108 FE analyses for bilayer implant and 1 FE analysis for conventional implant) to investigate the mechanical response of bone-dental implant. For all cases, the results have been analyzed in terms of von Mises stress distribution in the implant shell and the inner core and in terms of the strain distribution at the interface between the implant shell and cancellous bone.

## Results

### Strain and stress distributions in conventional implant

**Fig. 3:**
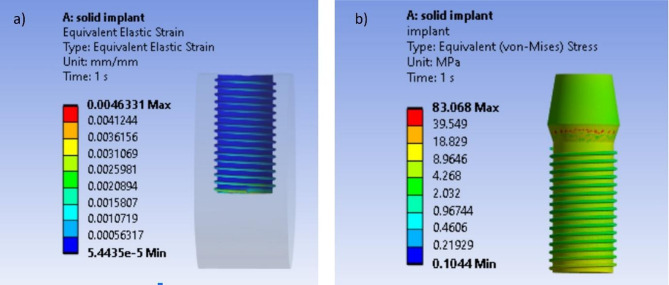
Strain and von Mises stress distributions for conventional implant (MPa). (**a**) Interfacial strain between surrounding bone and implant (MPa). (**b**) von Mises stress distribution.

Figure 3 shows the strain distribution at the interface between the conventional implant and surrounding bone (Fig. 3a) and von Mises stress distribution in the conventional implant (Fig. 3b). The maximum value of the von Mises stress for the conventional implant has been found to be equal to 83 MPa assessed at an average biting force of 250 N.

### Stress induced in bilayer implants with and without porosity

**Fig. 4:**
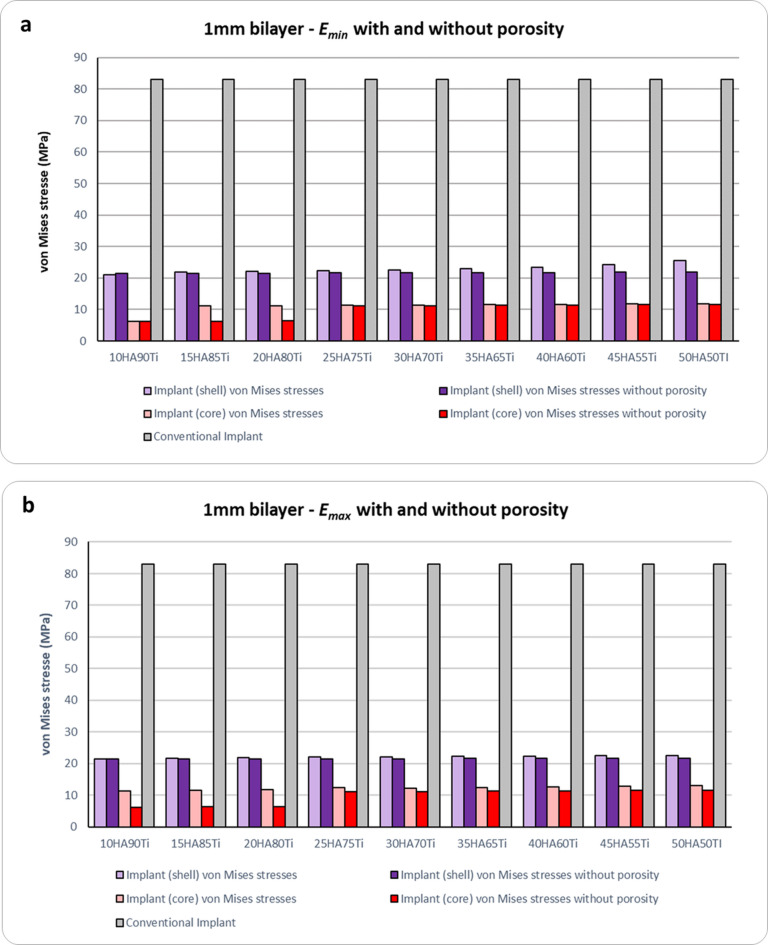
Von Mises stresses (MPa) induced in 1 mm bilayer implant, both with and without porosity, with different % of HA and Ti: (**a**) ${E}_{min}$; (**b**) ${E}_{max}$ .

**Fig. 5:**
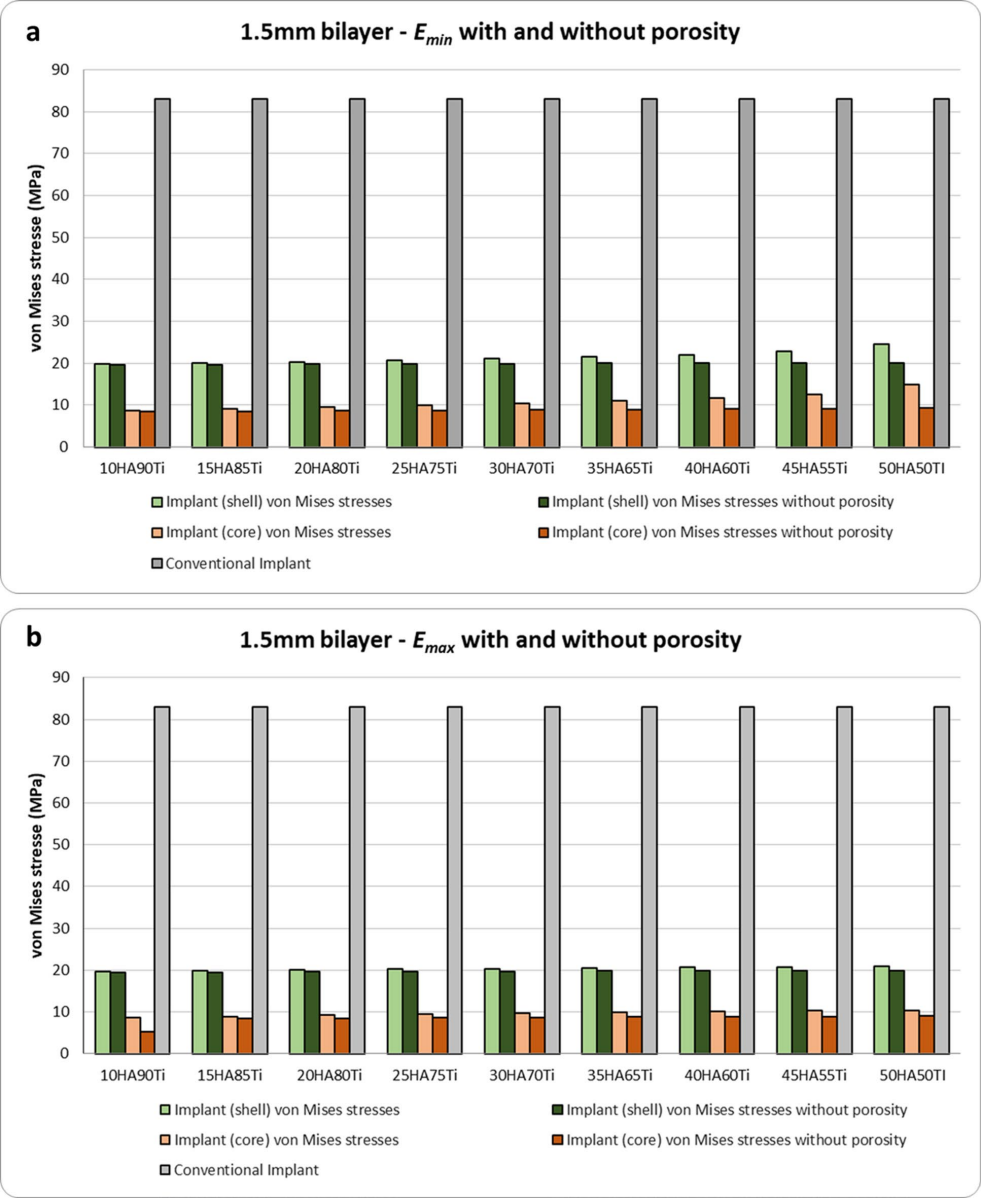
Von Mises stresses (MPa) in 1.5 mm bilayer implant, both with and without porosity, with different % of HA and Ti: (**a**) ${E}_{min}$; (**b**) ${E}_{max}$.

**Fig. 6:**
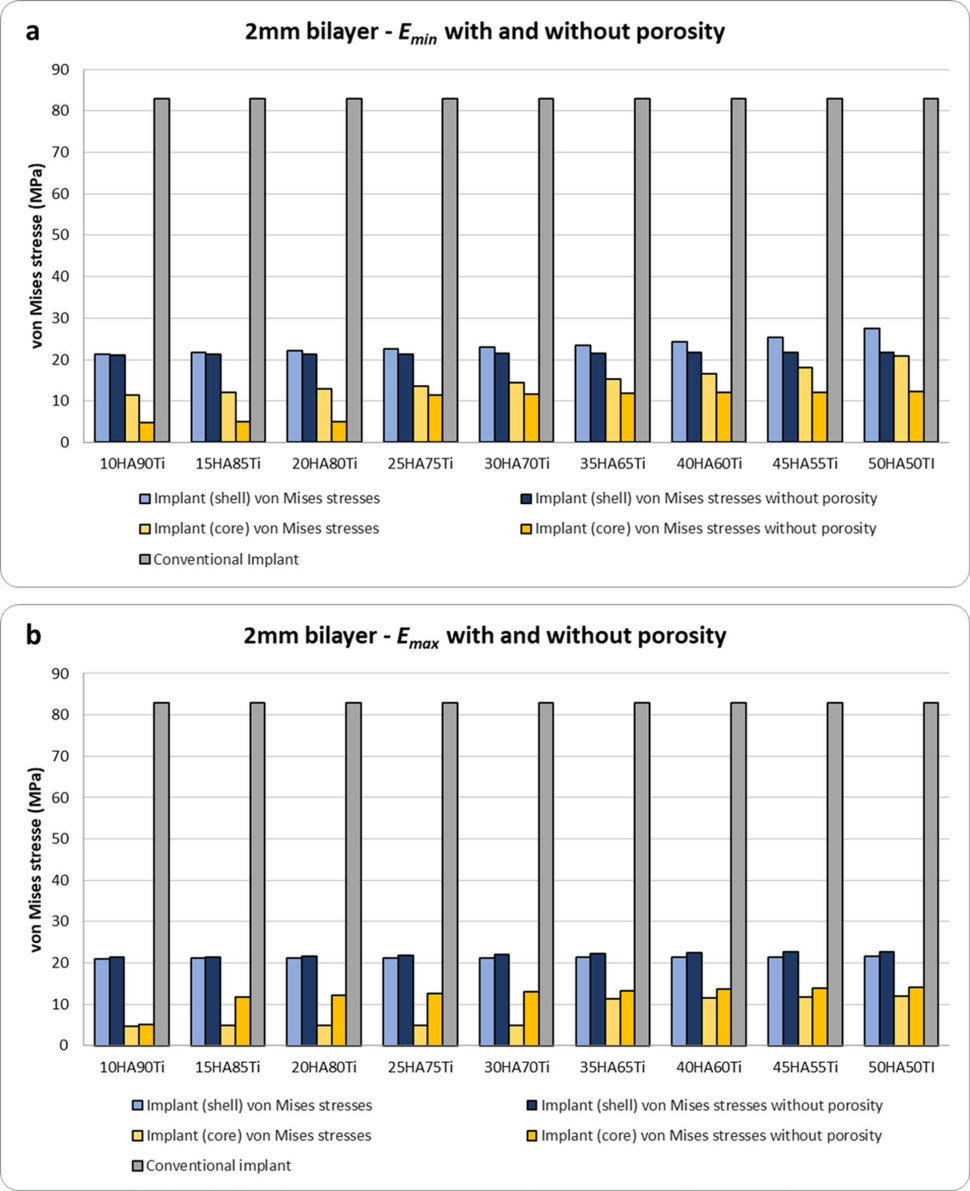
Von Mises stresses (MPa) in 2 mm bilayer implant, both with and without porosity, with different % of HA and Ti: (**a**) ${E}_{min}$; (**b**) ${E}_{max}$.

The von Mises stress results obtained for bilayer dental implants for ${E}_{max}$, ${E}_{min}$, ${E}_{max,p}$ and ${E}_{min,p}$ are shown in Figs. 4, 5 and 6. The HA concentration ranges from 10 to 50%, whereas the Ti content decreases from 90 to 50% (as shown in Table 2). The results show that in all cases the bilayer implants exhibit significant lower stresses compared to the conventional implant (Figs. 4, 5 and 6). Compared with the conventional implant (83 MPa), the porous designs exhibited decreases of approximately 69–77% in the shell and 84–94% in the core, depending on HA/Ti composition and shell thickness. Compared with the conventional implant (83 MPa), the non-porous bilayer configurations also showed clear stress reductions, with decreases of approximately 72–78% in the shell and 84–90% in the core, depending on HA/Ti composition and shell thickness. These reductions were lower than those observed in porous designs, with maximal reductions occurring in the porous core of some 2 mm bilayered implant configurations.

Figure 4a shows that, considering the upper bound of Young’s modulus for all combinations in terms of percentages of Ti and HA, a porous bilayer implant leads to an increase of the maximum stress in the shell compared to a non-porous bilayer implant. The maximum increase in stress for the shell is around 4% for a bilayer implant with 50% of HA and 50% of Ti. For the inner core, it can be observed that a porous bilayer implant increases the maximum stress with a significant increase when a low percentage of HA is considered (46% of increase for a bilayer implant characterized by 20% of HA and 80% of Ti). By increasing the percentage of HA, the rise in the maximum stress tends to reduce (10% of increase in the case of a bilayer implant with 50% of HA and 50% of Ti). A similar trend can be observed considering the lower bound of Young’s modulus (Fig. 4b).

Figure 5a shows that increasing the shell thickness a 1.5 mm bilayer implant characterized by ${E}_{max}$ tends to raise the maximum stress on the shell with respect to a non-porous implant (maximum increase around 5% for a bilayer implant with 50% of Ti and 50% of HA) and leads to increase the maximum stress on the inner core. For the inner core the rise in stress tends to decrease, increasing the percentage of HA (39% of increase for a bilayer implant with 10% of HA and 90% of Ti versus 10% of increasing for a bilayer implant with 30% of HA and 70% of Ti). Figure 5b shows a similar behavior for the shell when ${E}_{min}$ is considered. However, for the inner core it can be observed that a porous implant increases the maximum stress, and the rise becomes more significant when a greater percentage of HA is considered.

It is noteworthy that increasing the shell thickness, specifically to 2 mm, when ${E}_{min}$ is considered, results in a porous implant that increases the maximum stress in both the shell and inner core compared to a non-porous implant (Fig. 6a). However, when ${E}_{max}$ is considered, there is a significant decrease in the maximum stress in both the shell and inner core of a porous bilayer implant compared to a non-porous implant design in absolute terms (Fig. 6b). This is significantly higher in percentage terms for the inner core: increasing the %HA until 30 and the %Ti until 70, with the maximum decrease in stress reaching 61%.

### Interfacial strain in peri-implant bone

**Fig. 7:**
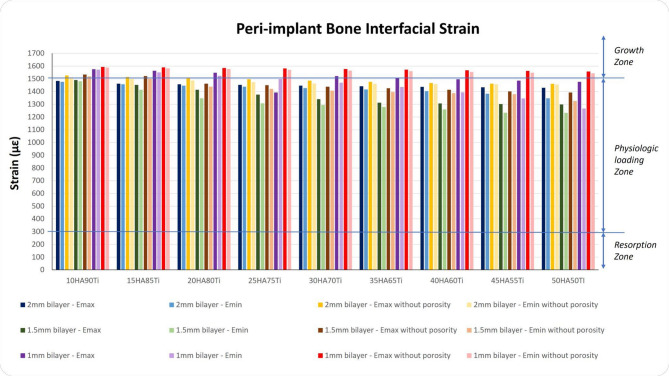
Interfacial strains on peri-implant bone for all bilayer implants tested, both with and without porosity (${E}_{min}$ and ${E}_{max}$). On the y axis right, three out of four windows of bone adaptation according to the mechanostatic theory with the zones’ mean threshold values^40,41^. με: microstrain.

The strains at the interface between the outer shell of the implant and the peri-implant bone have been quantified for all models and both the ${E}_{max}$ and the ${E}_{min}$ (Fig. 7). For all diameters of the inner core and for both the ${E}_{max}$ and the ${E}_{min}$, the porous implants show slight lower interfacial strains compared to the corresponding non-porous implants with values of interfacial strains located in the physiological range of Frost’s mechanostatic theory^5,40–42^. Moreover, for diameters of 2 and 3 mm of the inner core, the interfacial strains of porous and non-porous implants are lower than 1500 $\mu \varepsilon$ whereas for porous implants with an inner core of 4 mm in diameter with percentages of HA equal to 10%, 15% and 20% the interfacial strains result greater than 1500 $\mu \varepsilon$. Compared to the bilayer implants, the conventional implant shows a higher interfacial strain, i.e. 4633 $\mu \varepsilon$. This value corresponds to the overloading region of the mechanostatic theory.

It is important to highlight that lower strain values at the bone-implant interface are essential to reduce the risk of mechanical phenomena that may induce the implant failure.

### Strain and von Mises stress distributions in bilayer dental implant

**Fig. 8:**
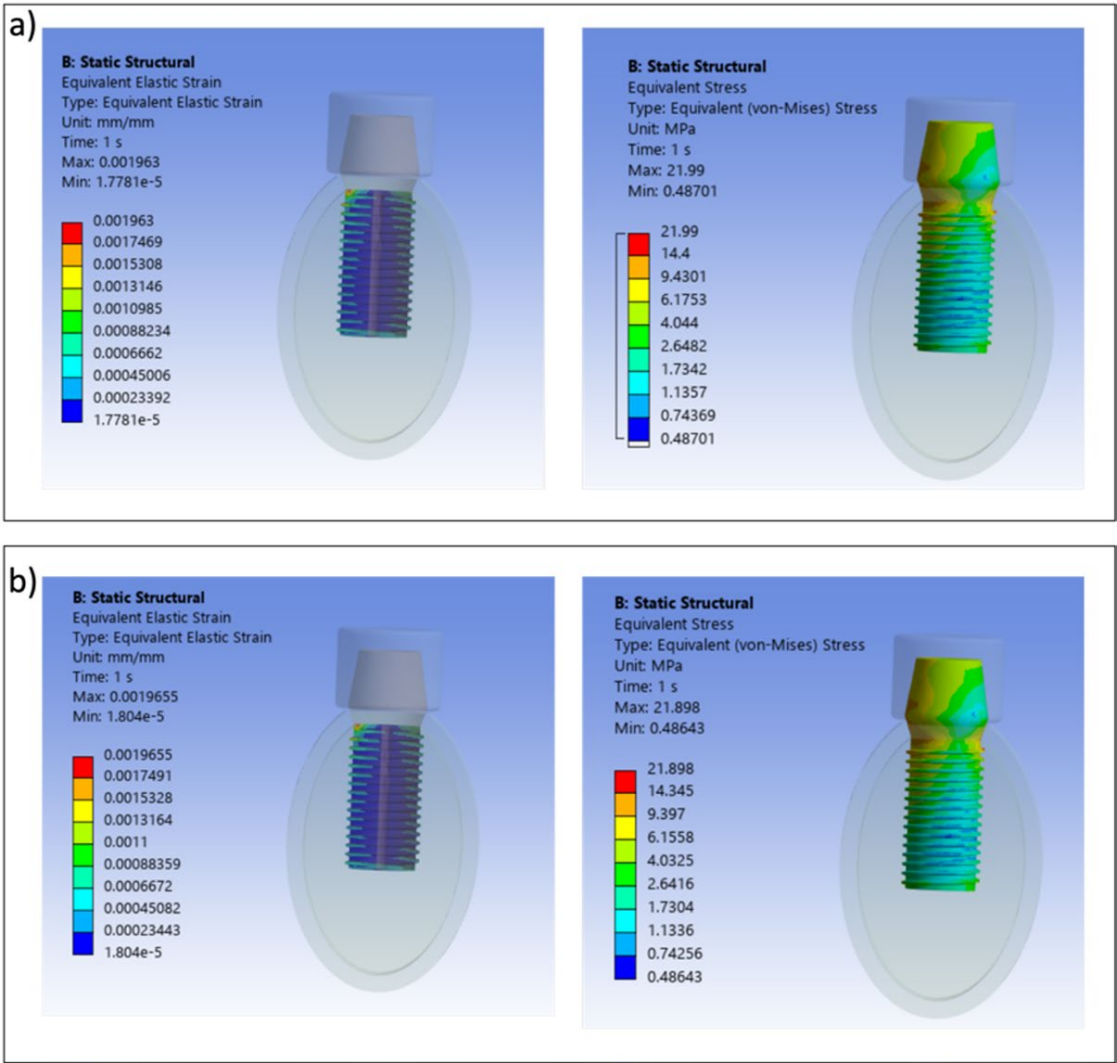
Interfacial strain and von Mises stress (MPa) distributions for porous 1 mm bilayer implant for 10HA90Ti configuration. (**a**) ${E}_{max}$; (**b**) ${E}_{min}$.

**Fig. 9:**
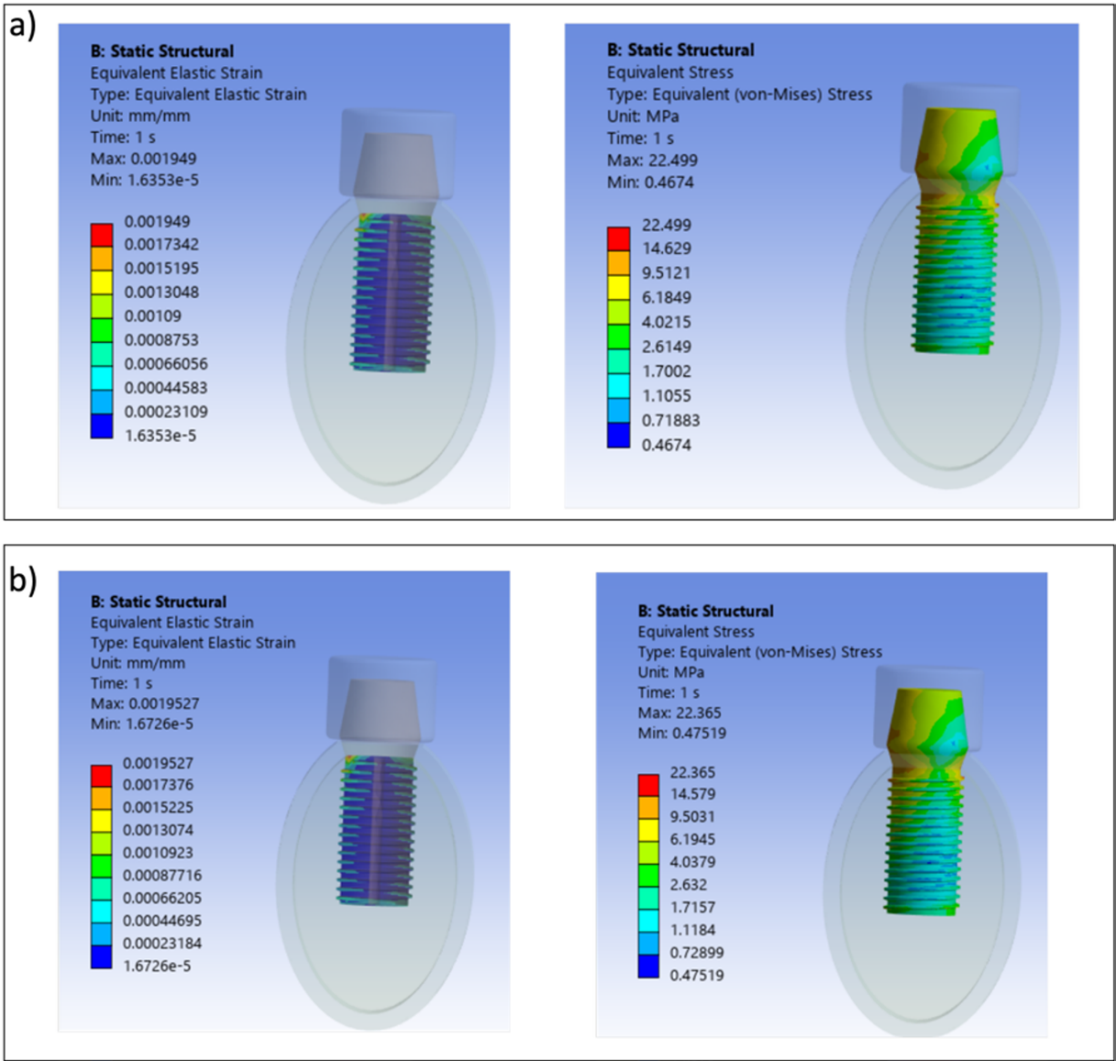
Interfacial strain and von Mises stress (MPa) distributions for 1 mm bilayer implant without porosity for 10HA90Ti configuration. (**a**) ${E}_{max}$; (**b**) ${E}_{min}$.

**Fig. 10:**
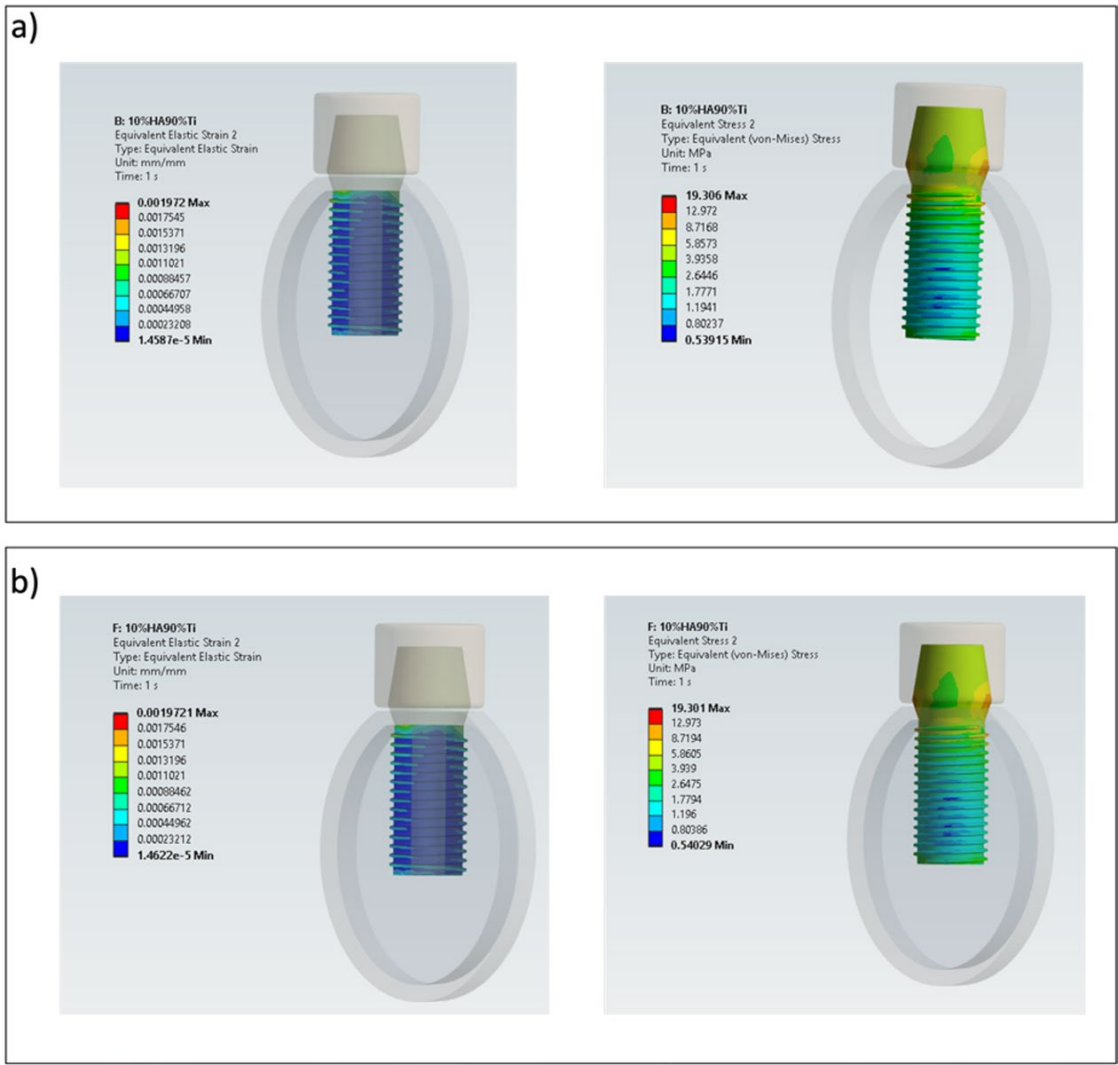
Interfacial strain and von Mises stress (MPa) distributions for porous 1.5 mm bilayer implant for 10HA90Ti configuration. (**a**) ${E}_{max}$; (**b**) ${E}_{min}$.

**Fig. 11:**
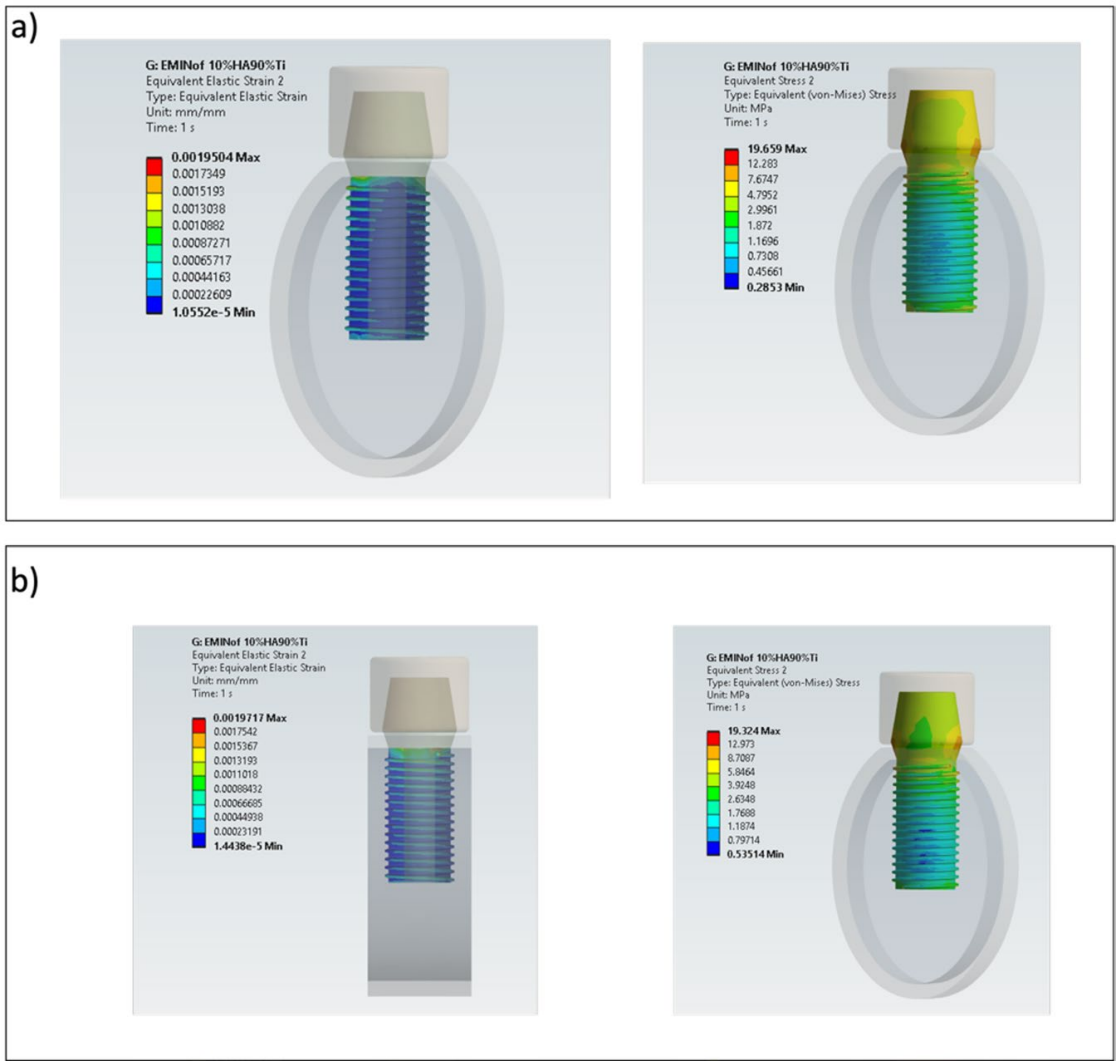
Interfacial strain and von Mises stress (MPa) distributions for 1.5 mm bilayer implant without porosity for 10HA90Ti configuration. (**a**) ${E}_{max}$; (**b**) ${E}_{min}$.

**Fig. 12:**
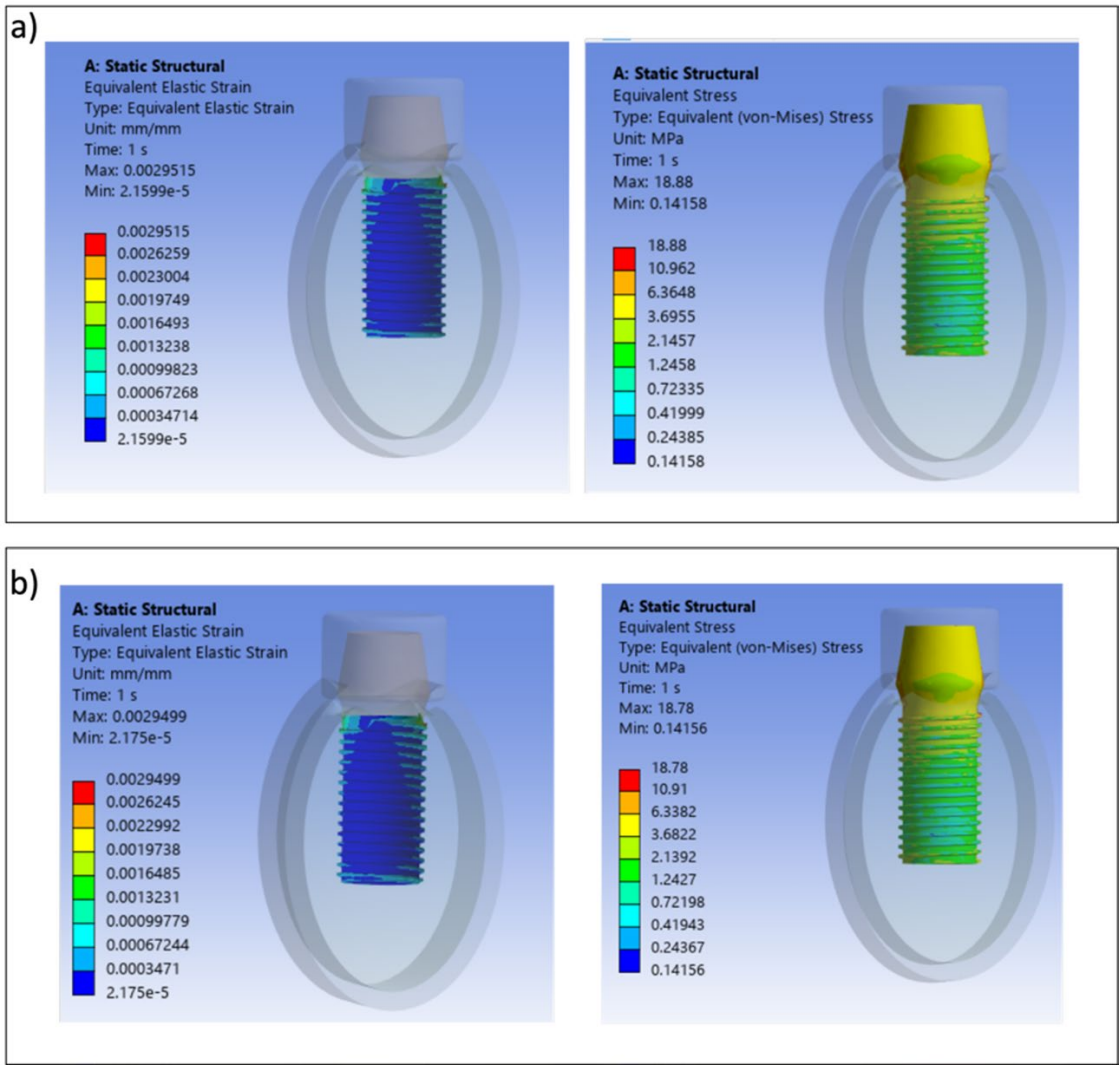
Interfacial strain and von Mises stress (MPa) distributions for porous 2 mm bilayer implant for 10HA90Ti configuration. (**a**) ${E}_{max}$; (**b**) ${E}_{min}$.

**Fig. 13:**
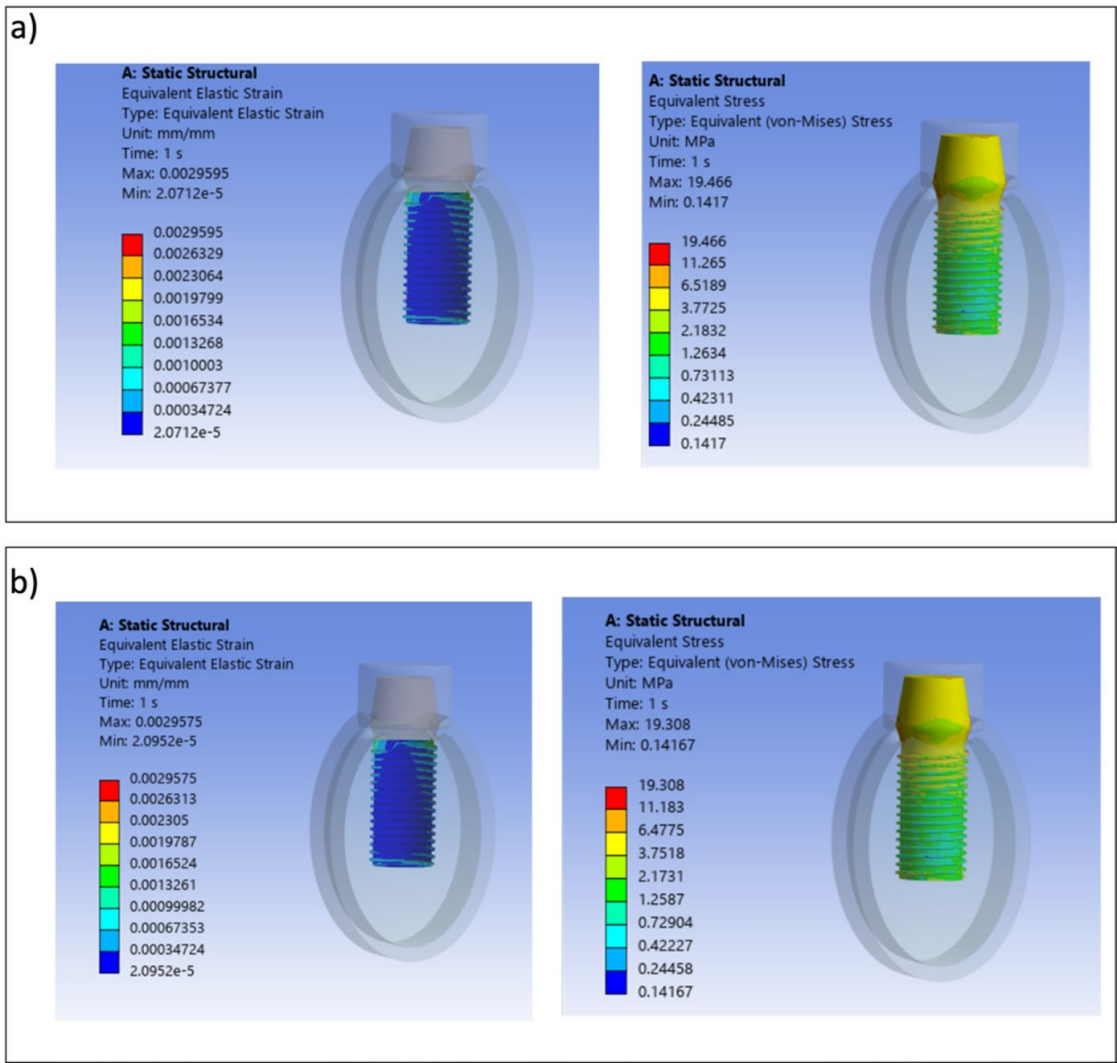
Interfacial strain and von Mises stress (MPa) distributions for 2 mm bilayer implant without porosity for 10HA90Ti configuration. (**a**) ${E}_{max}$; (**b**) ${E}_{min}$.

Von Mises stress and strain distributions are showed in Figs. 8, 9, 10, 11, 12 and 13 for the 10HA90Ti configuration, both with and without porosity and for all external shell thickness values T_L_. This configuration was chosen because the analyses reported in Figs. 4, 5, 6 and 7 revealed that it exhibited the most favorable mechanical response among all the investigated compositions. In fact, the 10HA90Ti bilayer implant provided an optimal balance between stiffness and stress transfer: the shell showed sufficiently low stress levels to prevent failure while ensuring efficient load transmission to the underlying bone, whereas the core maintained moderate stress values, indicating adequate mechanical support and preventing excessive stress shielding. Additionally, this configuration produced interfacial strain values within the physiological range of Frost’s mechanostatic theory, confirming its ability to mimic the biomechanical environment of natural bone. All bilayer designs demonstrated reduced stress and strain values compared to the conventional implant, with the porous variants consistently exhibiting slightly lower values than their non-porous counterparts. Among the three shell thicknesses, the 2 mm configuration showed the lowest peak stress while maintaining an interfacial strain distribution comparable to those observed for the other thickness values.

## Discussion

The objective of the present work was to develop an innovative implant design that could fulfill the increasing demand for advanced materials and designs, which would enhance implant stability and optimize patient outcomes. Therefore, a comparative examination was conducted to evaluate various combinations of porosity and HA content in a bilayered implant. The aim was to determine the optimal combination that could balance the biomechanical drawback of HA high stiffness with that of a porous structure’s lower stiffness, while also leveraging their respective biological benefits, including osteoconductivity and a bioactive surface.

In this context, porous biomaterials are widely recognized as osteoconductive agents, as new bone forms through creeping substitution from adjacent living bone. Porous structures, mimicking bone morphology, structure, and function, facilitate bone tissue formation by enabling osteogenic cell migration and proliferation, as well as vascularization^43^. In addition, HA is one of the most used materials to enhance dental implant surfaces, due to its excellent biocompatibility and osteoconductive behaviour^44^.

In the present work, the different implant configurations were analyzed in terms of von Mises stress and interfacial strain distributions. It is important to highlight that the interfacial strain must be interpreted in the context of Frost mechanostatic theory^40,41^, which states that bone remodels in response to mechanical stimuli. Strains within the physiological range provide an optimal stimulus for bone formation and adaptive remodeling. Strains below this range may lead to bone resorption, while strains above the overload threshold (around 4000 µε) can induce microdamage if sustained, highlighting the importance of implant designs that maintain peri-implant strains within this optimal window to support both bone health and implant longevity.

The analysis, performed with a maximum biting force of 250 N, also confirmed the von Mises stress values in the conventional implant by considering the known dimensions of the implant^30^. In a purely biomechanical and mathematical context, the microstrain levels detected (4633 µε) would invariably result in bone resorption, as they surpass the critical threshold of 4000 µε, which is the point at which the bone enters the overload window. However, the bone, being a dynamic and vital tissue, initiates a biological response that enables it to adapt and compensate for the excessive mechanical stress. This adaptive response is essential for maintaining bone integrity and function under conditions of mechanical overload.

For this reason, there is a requirement for the creation of bioactive multilayered titanium dental implants to promote accelerated growth of mechanically competent bone, as opposed to conventional solid dental implants^25^. From a biomechanical point of view, the best combinations of shell thickness, %HA%Ti and porosity that fall within the physiological range are crucial, as they trigger a maintenance bone response that aligns with homeostatic context. At the same time, the combinations that exceed 1500 µε are equally important, because over this level there is the most desirable window of microstrain for peri-implant bone (1500–2500 µε), as it promotes growth and adaptation to functional demands (i.e. corticalization) of the peri-implant bone, thereby increasing its biomechanical competence. This reduces the possibility of incurring the stress shielding effect^13^, or of falling into the overloading zone.

In this study, the porosity was selected based on literature recommendations to promote bone formation, as described in the Methods Section. However, the design of the porous structure should consider not only the optimization of bone ingrowth but also the risk of excessive fluid penetration from the oral cavity, which could induce inflammatory processes during bone remodeling. Previous works have indicated that pore sizes between approximately 200 μm and 1500 μm can effectively enhance bone ingrowth^36–38^, while allowing sufficient fluid exchange. Although specific evidence regarding the infiltration of oral fluids into highly porous dental implants is still limited, some of these studies suggest that pore dimensions within this range maintain a balance between nutrient transport and the prevention of excessive fluid penetration. Our results support that the selected porosity range provides favorable conditions for bone ingrowth while potentially reducing the risk of inflammation during the bone remodeling phase.

The observed optimal performance of the 2 mm bilayer porous implant with 10% HA and 90% Ti can be attributed to its ability to more closely mimic the mechanical properties of natural bone compared to other configurations. The inclusion of a porous outer layer with this specific HA/Ti ratio provides a balanced stiffness reducing stress shielding while providing adequate mechanical support. Experimental findings on immediately loaded conventional implants confirm that functionalization of the bone and the bone collagen fibres occurs when the bone strain is properly managed^45,46^. Therefore, the preferable implant design identified in the present study is the 2 mm bilayer porous implant with 10HA90Ti. This configuration is the only one that simultaneously reduces the maximum stress on the dental implant compared to a non-porous design, mitigates the risk of stress shielding, and induces physiological microstrain levels in the surrounding bone that promote bone remodeling and osseointegration. This dual effect is beneficial for both the homeostatic maintenance of the bone and its mechanical adaptive response. By ensuring optimal stress distribution and promoting healthy microstrain levels, this setup supports bone integrity and adaptability. Increasing the HA content beyond 10% was found to negatively impact mechanical performance. A higher proportion of HA tends to increase the overall modulus of the composite, resulting in increased stiffness mismatch with bone and a higher risk of implant failure due to reduced load transfer and potential microcracking. The value of 2 mm for the porous shell thickness falls within the range commonly reported for porous implants in various applications. For femoral stem implants, Mehboob et al.^47^ demonstrated that porous shells of 1.5–2.0 mm provided optimal performance across a wide porosity range (18–90%), whereas 1.0 mm shells were sufficient only at lower porosities and 0.5 mm shells only at very low porosities. Although that study focused on hip stems rather than dental implants, the underlying rationale remains comparable: in both cases, an adequately thick, porous shell promotes bone ingrowth and ensures long-term stability. It is also important to note that all the configurations containing 10HA90Ti warrant further investigation, as they consistently register at the 1500 µε level, which is a noteworthy achievement. The uniformity observed in these configurations indicates a significant potential, and therefore, it is essential to delve deeper into their properties and behaviors to fully understand their implications. This consistency suggests that the specific HA/Ti ratio plays a crucial role in achieving the desired mechanical environment for bone remodeling and adaptation.

From a clinical perspective, the proposed bilayered implant may enhance early-stage osteointegration combining the mechanical stability with enhanced biological activity. The porous Ti shell facilitates bone ingrowth and increases the surface area for improving the mechanical stability, while the inclusion of HA promotes cellular adhesion and osteoconductivity. These combined effects may lead to a faster bone-to-implant integration, potentially reducing healing time and improving implant stability during the critical post-operative period.

Nevertheless, the manufacturing process of the proposed bilayered implant presents certain challenges, particularly at the HA–Ti interface. The sintering of HA onto a Ti substrate may result in poor adhesion due to mismatched thermal expansion coefficients, which can induce residual stresses or microcracks. Additionally, high sintering temperatures may lead to HA decomposition, thereby reducing its bioactivity and long-term performance. To overcome these limitations, alternative fabrication techniques such as plasma spraying or low-temperature coating methods may be considered to ensure both mechanical integrity and biological functionality of the implant.

This study has some limitations. First, bone has been assumed to be homogeneous in terms of Young’s modulus, which may not accurately represent the natural heterogeneity of bone. Second, only one loading condition was explored, so it is necessary to consider several loading scenarios to fully understand the implant’s performance. Third, there is a lack of experimental validation to support the computational findings. Given the importance of validation in any modelling framework, the present study should be considered a proof-of-concept aimed at exploring the potential of bilayered porous implants to improve stability and bone integration. Future work will involve a comprehensive validation to assess the accuracy, robustness, and practical applicability of the implant. Fourth, the model only includes a portion of the bone rather than the entire mandible, which could influence the results. Fifth, this study did not directly assess the potential impact of fluid infiltration on inflammatory processes during bone remodeling. Future work should include investigations to clarify the relationship between pore size, porosity percentage, and oral fluid infiltration, with the aim of further optimizing implant design for both biological performance and long-term stability. Lastly, the present study offers a limited evaluation of mechanical performance, particularly under physiological shear and compressive forces. The mechanical stability of the bilayered implant remains a crucial concern, with specific attention needed on the influence of HA layer thickness. While a thicker HA shell may enhance biological integration, it could also compromise mechanical integrity due to the material’s inherent brittleness, especially under lateral shear and longitudinal loading. As such, future work should include FE analyses to assess whether stress distributions remain within acceptable limits. Additionally, experimental validation through mechanical testing, such as compression, shear, and fatigue protocols, will be essential to confirm the implant’s load-bearing capacity and long-term reliability.

To improve the study, future research should address the heterogeneity of bone deriving the heterogeneous Young’s modulus distribution from diagnostic images such as Computed Tomography images. It would also be beneficial to test the STL file of existing implant geometries with different types of connections. Further research is essential to fully realize the benefits of this innovative implant design in clinical settings. This involves studying the impact of porous HA implants on stem cells during bone regeneration, which will help assess their effectiveness in reconstructing the mandibular bone and ensuring successful integration with the surrounding tissue^48^. Understanding the biological interactions at the cellular level can provide insights into how the implant material and structure influence bone healing and long-term stability. Notably, the enhanced interaction with human osteoblasts, gingival fibroblasts, mesenchymal stem cells, and monocytes suggests that the 3D-printed porous implant promotes superior biological responses than machined conventional implant, indicating its potential for future clinical applications^49^.

Moreover, considering that the most effective biomechanical implant in this study was the 2 mm bilayer implant, featuring a 2 mm inner titanium core and an outer porous shell of 10%HA and 90%Ti with 10% porosity, future research should explore how the biomechanical behavior of this implant changes by maintaining the composition of the outer layer unaltered and varying the porosity. Adjusting the porosity could fine-tune the balance between mechanical strength and biological integration, potentially leading to even better performance. Finally, using 3D printing technology to manufacture the implants could provide precise control over its structure, enabling tailored designs that fit individual patient needs. Additionally, applying topological optimization can minimize material usage, leading to more sustainable production methods.

## Conclusion

The study demonstrates that the innovative bilayered dental implant design, characterized by a Ti alloy core and a porous outer layer incorporating HA, offers superior biomechanical performance compared to conventional solid implants. The results indicate that the optimal configuration is the 2 mm bilayer implant, with 2 mm inner titanium core and 10%HA and 90%Ti in the outer porous shell, as it exhibits favorable biomechanical features. The balanced HA content and optimized porosity significantly reduced von Mises stress values and improved stress distribution at the bone-implant interface. The findings underscore the potential of bilayer porous implants in improving implant stability and patient outcomes.

## Data Availability

The datasets generated during and/or analysed during the current study are available from V.K.D. and S.R. on reasonable request.
